# INFLUENCE OF DEPRESSION, SOCIAL SUPPORT AND MEANING IN LIFE ON SUICIDAL IDEATION OF OLD ADULTS HEMODIALYSIS PATIENTS

**DOI:** 10.1093/geroni/igz038.3245

**Published:** 2019-11-08

**Authors:** Hyebeen Sim, Jinhee Shin

**Affiliations:** 1 Kyung Hee university, Kangbuk samsung hospital, Seoul, Korea, Republic of; 2 College of Nursing, Yonsei University, SOUTH KOREA, SEOUL, Korea, Republic of

## Abstract

Purpose: This study is to identify how depression, social support and meaning in life influence to suicidal ideation of home based Korean old adult renal dialysis patients and the relating factors according to their general characteristics. Methods: This descriptive correlative study was conducted through a organized and structured self-administrated questionnaire and 120 sampled home based old adult renal dialysis patients. Collected data was analyzed by t-tests, ANOVA, Pearson’s correlation coefficient and multiple regression analysis using SPSS/WIN 24.0. Results: Findings revealed that; 1) The degrees of suicidal ideation were significantly different among groups according to the marital status, drinking and smoking history. 2) Pearson’s correlation coefficient revealed a significant association among the suicidal ideation, depression, social support and meaning in life. 3) Multiple regression analysis showed depression, social support and meaning in life were related to factors. Conclusion: Based on the findings of this study, health professionals should provide home based Korean old adult renal dialysis patients with proper management of suicidal ideation as well as its relating factors, depression, social support and meaning in life. Especially, it needs to implement suicidal ideation management and self-help group program to home based old adult renal dialysis patients.

